# Clinical Management of the Microbiome in Irritable Bowel Syndrome

**DOI:** 10.1093/jcag/gwz037

**Published:** 2020-01-04

**Authors:** Christopher N Andrews, Sacha Sidani, John K Marshall

**Affiliations:** 1 Department of Medicine, Cumming School of Medicine, University of Calgary, Calgary, Alberta, Canada; 2 Centre hospitalier de l’Université de Montréal, Montreal, Quebec, Canada; 3 Department of Medicine and Farncombe Family Digestive Health Research Institute, McMaster University, Hamilton, Ontario, Canada

**Keywords:** *Constipation*, *Diarrhea*, *Irritable bowel syndrome*, *Microbiome*, *Prebiotics*, *Probiotics*

## Abstract

**Background:**

A growing body of evidence suggests that dysbiosis contributes to the onset and symptomatology of irritable bowel syndrome (IBS) and other functional bowel disorders. Changes to the gastrointestinal microbiome may contribute to the underlying pathophysiology of IBS.

**Methods:**

The present review summarizes the potential effects of microbiome changes on GI transit, intestinal barrier function, immune dysregulation and inflammation, gut–brain interactions and neuropsychiatric function.

**Results:**

A multimodal approach to IBS management is recommended in accordance with current Canadian guidelines. Pharmacologic treatments are advised to target the presumed underlying pathophysiological mechanism, such as dysregulation of GI transit, peristalsis, intestinal barrier function and pain signalling. The management plan for IBS may also include treatments directed at dysbiosis, including dietary modification and use of probiotics, which may promote the growth of beneficial bacteria, affect intestinal gas production and modulate the immune response; and the administration of periodic short courses of a nonsystemic antibiotic such as rifaximin, which may re-establish microbiota diversity and improve IBS symptoms.

**Conclusion:**

Dysregulated host–microbiome interactions are complex and the use of microbiome-directed therapies will necessarily be empiric in individual patients. A management algorithm comprising microbiome- and nonmicrobiome-directed therapies is proposed.

## Introduction

The gastrointestinal (GI) microbiome comprises the organisms, including bacteria, viruses, fungi and archaea that reside along the length of the GI tract and interact with the host. Alterations to the complex host–microbiome ecology, such as changes in the type or quantity of micro-organisms or their interactions with the host immune system, are termed dysbiosis (1). As such, dysbiosis does not represent a single mechanism. Rather, it describes a dysregulation of what has been termed the ‘microbiota organ’ (1). This may include loss of microbial diversity, imbalance in the relative proportion of pathogenic and beneficial micro-organisms, or dysregulation of metabolic products (2–4). As with other organ systems, alterations in the structure and function of the microbiome will affect and be affected by the host’s genetics, sex, aging, immune function and environmental factors, such as diet and antibiotic exposure (5–7). Dysregulation of the microbiome may manifest clinically as disorders of the GI tract, as well as of distant organs, such as the liver, pancreas and brain (8,9).

There is emerging evidence to suggest that dysbiosis contributes to the onset and symptomatology of irritable bowel syndrome (IBS) and other functional bowel disorders (10). While research is still in its preliminary stages, this line of inquiry has the potential to categorize IBS patients according the subtype of microbiome dysfunction and may lead to a more individualized treatment approach. This review summarizes the alterations in microbiome composition and function that may contribute to the pathophysiology of IBS and proposes a rational approach to managing dysbiosis to improve clinical outcomes.

## Gut Microbiome

The Human Microbiome Project characterizes humans as ‘supraorganisms’, or composites of the combined genomes of the host and the micro-organisms that live on or in the host (11). These micro-organisms outnumber human cells by a factor of 10. There are approximately 20,000 protein-coding genes that make up the human genome. The coding capacity of the microbial genome is estimated to be 150-fold higher (1), which serves to extend the range of traits or functions that the human genome has not evolved (11). These functions include the breakdown of dietary carbohydrates and proteins, production of short-chain fatty acids (SCFA, e.g., butyrate, propionate, acetate), synthesis of vitamins and amino acids, production of bile acids, modulation of lipid metabolism and storage, and detoxification of carcinogenic compounds (12).

Most of the human microbiome is composed of organisms residing in the GI tract. The number and composition of microbiota lie along a continuum, with an estimated 10 to 1000 bacteria/gram in the stomach and duodenum, 10^4^ to 10^7^ bacteria/gram in the jejunum and ileum, and 10^11^ to 10^12^ bacteria/gram in the colon (10,13). Two phyla, Firmicutes and Bacteroidetes, make up about 90% of the bacterial microbiome (12). The predominant genera in the intestinal lumen differ from those in the intestinal mucosa (14), a consideration when interpreting the results of fecal sampling.

Microbial exposure begins in utero or during childbirth, with further microbiota expansion and diversification with exposure to food and the environment during early development (15,16). The microbiome composition is affected by the method of birth (vaginal, Caesarean section) and the type of feeding (breast, formula) (17,18). Many factors will influence which organisms are able to colonize the various ecological niches along the GI tract, such as intestinal function (e.g., acid production, peristalsis, transit time, barrier function), nutrition, the timing of microbial acquisition, and species adaptability in light of competitive pressures and the development of the host immune response. Early colonizing species are typically aerobic organisms (e.g., *Staphylococcus, Streptococcus*), whereas late colonizers are generally anaerobes (e.g., *Clostridium*) (10).

The acquisition of the gut microbiome occurs in concert with the development of the infant’s innate and adaptive immune systems. Colonizing bacteria are the first antigens, which serve to activate the immature immune response, promote intestinal barrier function, establish immune tolerance and influence the infant’s response to potential allergens (19). Immune effects of colonizing bacteria include activation of immunoglobulin-A (IgA), and interaction with Toll-like receptors to activate effector cells, such as macrophages, B cells and T-helper (Th1, Th2) cells. Also stimulated are regulatory T cells (Treg), which are required for immune tolerance (20) and which have been implicated in the development of allergy, autoimmunity and chronic inflammatory conditions (21). These findings suggest that dysregulation of host–microbiome interactions may play a key role in the etiopathogenesis of IBS.

## Dysbiosis in IBS

IBS is a common GI disorder with an estimated prevalence in Canada of 6 to 12% depending on the criteria used (22,23). According to the most recent guidelines by the Canadian Association of Gastroenterology (CAG), the recommended diagnostic criteria for IBS are the Rome IV criteria, which require abdominal pain (≥1 day/week for ≥3 months) associated with defecation or a change in bowel habits (24). Subtypes are constipation-predominant (IBS-C; >25% hard stools, <25% loose stools), diarrhea-predominant (IBS-D; >25% loose stools, <25% hard stools), mixed (IBS-M; >25% loose stools, >25% hard stools) and unclassified (IBS-U; <25% loose stools, <25% hard stools).

There are conflicting data as to whether there are alterations in the number or relative proportion of bacterial enterotypes in IBS. A *Prevotella*-dominant enterotype has been negatively associated with IBS symptom severity (25); positively associated with IBS-D (26); and found to be no different in its expression in IBS versus healthy controls (27). Similarly, an increased Firmicutes-to-Bacteroidetes ratio has been reported in some but not all studies (28,29). In part, these inconsistent findings may be because the composition of the microbiome in eubiosis and dysbiosis has not been determined. There may also be differing results due to the populations studied (e.g., IBS subtype), how and in what part of the GI tract the samples were obtained, the molecular techniques used, or other factors (10,30).

The microbiome is generally stable in adulthood but can be perturbed following an acute GI infection, the most common risk factor for the development of IBS. The estimated prevalence of post-infection IBS (PI-IBS) is 4 to 32% (31,32). Changes to the microbiota in PI-IBS, such as depletion of butyrate-producing bacterial strains, suggest that similar alterations may occur in IBS (33,34). The use of broad-spectrum, systemic antibiotics is also associated with an increased risk of IBS and non-IBS bowel symptoms (35,36), but the effect of different antibiotics on microbiota depletion has not been extensively studied.

More controversial is the association between IBS and small intestinal bacterial overgrowth (SIBO) (reviewed in ref. (37)). A recent meta-analysis reported a higher prevalence of SIBO in IBS patients versus controls (odds ratio 4.7) (38). Possible microbiota changes with SIBO include an increase in methane-producing bacteria, which may be associated with IBS symptoms such as abdominal distension, pain and flatulence (39); and an increase in sulfate-producing strains, which may be associated with visceral hypersensitivity (40). However, accurate diagnosis of SIBO is a challenge (reviewed in [41]). The most common approach, hydrogen breath testing using glucose or lactulose substrate, has poor specificity and may be a more accurate measure of transit time than bacterial counts in the small intestine (42). Hydrogen and methane-based breath testing may be obtained according to current consensus recommendations (43), however, the recent CAG guidelines suggest against performing hydrogen breath testing in patients with IBS (24). Stool cultures have little value since the microbiome of the small intestine differs greatly from that of the colon (14).

Numerous pathophysiological mechanisms may contribute to the development of IBS, notably gut dysmotility, changes in intestinal barrier permeability, immune activation and inflammation, abnormal brain–gut interactions, hypersensitivity to visceral stimuli and psychological stressors (10). Each of these mechanisms may be associated with specific changes to the microbiome, although it is more likely that the complex interactions among various species, and how this ecology influences the host immune response, will ultimately better describe the genesis of symptoms in IBS and other bowel disorders. The following summarizes how the microbiome may contribute to varying degrees to the underlying mechanisms in IBS.

## Gastrointestinal Transit

GI transit time is inversely related to bacterial abundance and diversity, as suggested by the increasing bacterial count from the duodenum to the colon. Stool consistency is positively associated with the Bacteroides-Firmicutes ratio and the abundance of methane-producing species (e.g., *Methanobrevibacter smithii*) (44). Bacterial CH_4_ production has been associated with slower GI transit and constipation (25,45), with some data indicating that *M. smithii* is more abundant in IBS-C (46,47). Two studies have reported that the nonsystemic antibiotic rifaximin accelerated colon transit, which was associated with a reduction in CH_4_ production and improvements in stool frequency and consistency (48,49). A caveat is that CH_4_ production in clinical studies is generally estimated from breath methane testing, which may not accurately reflect CH_4_ production in the colon (50).

## Intestinal Barrier Function

Intestinal barrier dysfunction and increased permeability have been implicated primarily in IBS-D and PI-IBS (51). It has been reported that alterations in intestinal permeability in IBS-D are associated with decreased expression of tight-junction proteins such as zonula occludens-1 (ZO-1) and occludin; reduced occludin expression was correlated with duration of IBS symptoms and abdominal pain severity scores (52).

Commensal bacteria may have direct effects on the mucosal layer and the intestinal epithelial cells that make up the intestinal barrier. *Lactobacillus* have been shown to increase the expression of mucin in intestinal cell lines, blocking the adherence of bacteria (*Helicobacter pylori, Pseudomonas aeruginosa*), fungi (e.g., *Candida albicans*) and parasites (e.g., *Entamoeba histolytica*) that degrade mucus (51). Re-establishing commensal bacterial populations with probiotics can promote tight-junction and barrier repair (reviewed in ref. (53)). Some strains of adhesive lactobacilli and bifidobacteria have been shown in vitro to inhibit the adhesion of GI pathogens to intestinal cells through active mechanisms (reviewed in ref. (54)) and niche competition (55), and to inhibit cell invasion by pathogens (56). In vitro and animal models have indicated that commensals also have antimicrobial effects through the production of metabolites such as lactic acid, which has activity against *Helicobacter pylori* (57,58); bacteriocins, which inhibit colonization by *Clostridium difficile* (59), *Staphylococcus aureus* (60), *Listeria monocytogenes* (61), and vancomycin-resistant enterococci (62); and antimicrobial peptides (e.g., cathelicidin, defensins), which promote mucus synthesis and intestinal epithelial repair (63,64). Rifaximin has been shown to alter the attachment and internalization of pathogenic bacteria (65), which may be due in part to changes to the physiology of epithelial cells (66). In travellers’ diarrhea, rifaximin reduced the expression of bacterial virulence factors, an effect that appeared to be partially mediated by downregulation of matrix metalloproteinase (MMP)-9, an enzyme that degrades barrier function (67). In an animal model of colitis, rifaximin reduced bacterial translocation to mesenteric lymph nodes, which was associated with a reduction in proinflammatory cytokines (68). Some authors have suggested that impaired barrier function may not directly cause IBS symptoms, but may be associated with inflammation and altered sensorimotor function (69).

## Immune Dysregulation and Inflammation

Loss of barrier integrity may be associated with increased passage of commensal and pathogenic bacteria across the intestinal epithelium, resulting in immune activation and subclinical inflammation. Barrier translocation has been shown to be partly regulated by mast cells in IBS patients (70), although it is unclear if the number of mast cells is elevated in IBS or whether such increases are associated with symptoms (69). Of greater importance to IBS-related visceral hypersensitivity and abdominal pain are the pro-inflammatory mediators produced by mast cells (e.g., histamine, proteases, prostaglandins, serotonin), and the proximity of mast cells to enteric nerves (71–74).

Dysbiosis may also be associated with alterations in the innate immune response, notably increased expression of Toll-like receptors (e.g., TLR-4 and -5) by macrophages and the release of TLR-associated cytokines (e.g., interleukin-1β, IL-6, IL-8, tumour necrosis factor [TNF]-α) (75–77). Serum levels of IL-6, IL-8 and TNF-α have been proposed as an immune biomarker of IBS (78) but this requires further validation.

The adaptive immune response has been less studied in IBS, although a recent study found a significant increase in CD4+T cells expressing the gut-homing marker integrin β7 (79). In addition, there are data to suggest dysregulation of the normal balance of Th1/Th2 subsets, with IBS patients demonstrating elevated baseline levels of proinflammatory cytokines, such as IL-1β, TNF-α and IL-6 (80). This profile of elevated IL-1β and TNF-α has been linked to abdominal cramps, pain, nausea/vomiting and delayed gastric emptying in functional dyspepsia (81) and may be a factor in IBS symptomatology. Gene polymorphisms affecting Th cytokines have also been reported to be overrepresented in IBS patients compared to controls (82). An emerging area of interest is epigenetic changes to pro-inflammatory factors (e.g., NF-κB, hypoxia-inducible factor) by SCFAs such as butyrate, which promotes Treg differentiation (83–85). Butyrate has been shown in vitro to inhibit the pro-inflammatory response (Th1 response, interferon-γ production), an effect that is partially mitigated by acetate and propionate (86).

## Gut–Brain Axis and Neuropsychiatric Function

The concept of a gut–brain axis with bidirectional communication between the central and enteric nervous systems (CNS, ENS) was introduced almost four decades ago (87). Psychological factors (e.g., stress, depression, anxiety) have long been accepted as contributing to IBS. A recent meta-analysis found that levels of depression (standard mean difference 0.76) and anxiety (SMD 0.84) were higher in IBS patients compared to healthy controls (88). Chronic stress and depression are now known to alter the microbiota, increasing Escherichia coli*, Pseudomonas* and Enterobacteriaceae and decreasing Lactobacilli and Faecalibacteria (89,90).

Conversely, changes to the microbiota may affect neuropsychiatric function via several possible mechanisms. Microbiota produce a variety of neurotransmitters, such as norepinephrine (*Escherichia, Bacillus, Saccharomyces*), serotonin (*Escherichia, Streptococcus, Candida*), γ-amino butyric acid (GABA) (*Lactobacillus, Bifidobacterium*), acetylcholine (*Lactobacillus*) and dopamine (*Bacillus*) (91). *Lactobacillus* has been shown in animal studies to increase GABA mRNA expression in the brain, an effect not found in vagotomized mice (92). A small study in healthy women reported that probiotic consumption was associated with changes in connectivity to the midbrain, a region involved in visceral pain sensitivity and emotional processing (93). Also noteworthy is the elevated level of serotonin produced by mast cells and enterochromaffin cells in the GI tract of IBS patients, which has been significantly correlated with abdominal pain severity (64).

Studies of fecal microbiota transfer (FMT) from IBS patients to rodents have reported the development of IBS features (accelerated GI transit, intestinal barrier dysfunction, immune activation) and symptoms (anxiety, hypersensitivity to colonic distension) following transplantation (94,95). Microbiota changes included an increase in Enterobacteriaceae and a decrease in bifidobacteria. Recent authors have proposed a microbial signature of psychological distress in IBS based on an association of *Proteobacteria* abundance with anxiety, depression and stress perception; decreased Lachnospiraceae with depression and increased Bacteroidaceae with anxiety (96).

## Therapeutic Strategies

The current Canadian practice guidelines recommend a number of therapeutic strategies to manage IBS (24). Psychological therapies include cognitive-behavioural therapy and hypnotherapy. Medical therapies include antispasmodics (e.g., dicyclomine, hyoscine, pinaverium) and antidepressants (e.g., tricyclics, selective serotonin reuptake inhibitors). In addition, the guidelines acknowledge that treatments directed at dysbiosis may also be beneficial. Such therapies may include the following:

### Dietary Modification

There is very low-quality evidence to support a low FODMAP (fermentable oligosaccharides, disaccharides, monosaccharides, polyols) diet to alter colonic microbiota production of gases and SCFAs, and to improve abdominal pain, bloating, frequency and urgency in IBS (24). The evaluation of the evidence quality is based on the general difficulty of performing dietary studies. However, the full restriction phase of the diet is not recommended long-term and should be implemented with the guidance of a dietitian. Other recommended treatments are soluble fibre and psyllium, which provide bulk, are fermented to SCFAs and promote the growth of lactobacilli and bifidobacteria by lowering colonic pH (97). An elemental diet may also be beneficial in patients with bacterial overgrowth (98). Gluten-free diets and wheat bran supplementation do not appear to be helpful in the management of IBS.

### Probiotics/Prebiotics

Probiotics appear to improve global symptoms, abdominal pain, bloating and flatulence scores based on meta-analysis (24). Although the effects of probiotics are modest, the safety profile is very good (99). However, despite numerous studies, there are significant limitations to the evidence base, such as the use of widely variable strains or combinations of microbial species, and uncertainty as to the viability and constituents of commercial products due to a lack of regulations to ensure product quality (24).

Prebiotics do not appear to improve GI symptoms in IBS patients, according to a recent meta-analysis (98). There was no difference with prebiotics versus placebo with respect to abdominal pain, bloating and flatulence, although there was some improvement in flatulence severity.

### Antibiotics

Broad-spectrum antibiotics (e.g., amoxicillin, rifaximin, ciprofloxacin) are commonly used in SIBO (100), and a number of studies have investigated broad-spectrum antibiotics in IBS, primarily to target bacterial overgrowth. Neomycin has been shown to improve IBS symptom scores (101) but most patients fail to respond to retreatment (102). Similarly, retreatment is often ineffective with doxycycline, amoxicillin/clavulanate and ciprofloxacin, an effect attributed to early development of antibiotic resistance (102,103). A further concern is systemic adverse effects with these agents.

Rifaximin is a nonsystemic antibiotic that has been shown in multiple clinical trials to be effective in improving global IBS symptoms, bloating, abdominal pain and stool consistency (104,105). Rifaximin was recently approved for nonconstipation IBS in Canada, and is recommended for the treatment of IBS by the American College of Gastroenterology (106). A metagenomic analysis of patients with GI and liver diseases reported that rifaximin was associated with a significant increase in Lactobacilli without significant alterations in the overall gut ecology (107). A second analysis in nonconstipation IBS reported an increase in bacterial diversity with rifaximin, including a reduction in *Clostridium* and a shift in the Firmicutes/Bacteroidetes ratio (108). These changes in the composition of the microbiota may contribute to an anti-inflammatory effect at the level of the intestinal mucosa (106). Several authors have noted that rifaximin is an agonist of the pregnane X receptor (PXR) (109), which inhibits nuclear factor (NF)-κB and its transcription of proinflammatory cytokines, such as TNF-α. Rifaximin has been shown to downregulate NF-κB genes and improve recovery from colitis symptoms in PXR-humanized animal models but not in PXR-null mice (110), suggesting that the anti-inflammatory effect is mediated by PXR. In addition, rifaximin appears to have direct effects on bacterial metabolism, colonic methane production and the expression of virulence factors (48,111,112), as well as effects on host mucosal inflammation and bacterial attachment (112). Repeated courses of rifaximin do not appear to significantly alter the antibiotic sensitivity of microbiota (113).

### Fecal Microbiota Transplantation (FMT)

This approach is novel and not currently recommended outside of clinical trials. Two recent trials have reported conflicting results. A double-blind study in patients with moderate-to-severe IBS reported symptom relief (>75 points on the IBS Symptom Severity Score) in 65% of active-treatment patients versus 43% of controls (114). In contrast, a study involving a similar population found that FMT was effective in altering the microbiome but there were greater improvements in symptom and quality of life scores with placebo (115). These differences may be due to the patient populations, the study methodology or the microbial diversity or donor samples, which has been shown to be a predictor of successful transplantation (116).

## Clinical Management

IBS is currently managed according to the predominant symptoms of pain, diarrhea (IBS-D) or constipation (IBS-C), with pharmacologic treatments targeting the presumed underlying dysfunction, such as GI transit (e.g., loperamide, eluxadoline, laxatives), peristalsis (antispasmodics), epithelial ion channels (linaclotide) or pain signalling (e.g., tricyclic antidepressants). This approach is necessarily empiric since the underlying pathophysiology is unknown, may differ in subgroups of patients or may evolve over time in individual patients.

**Figure 1.**
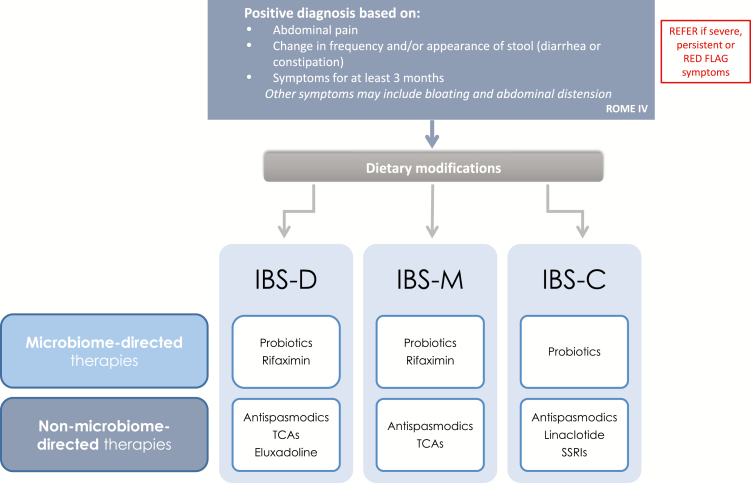
Proposed therapeutic approach to managing the microbiome in IBS. It should be noted that the actual management approach to any given IBS patient will depend on multiple factors; the therapeutic strategies shown should not imply that microbiome-related approaches should be tried before nonmicrobiome related therapies. A multimodal approach comprising dietary/lifestyle, medical, and/or psychological therapies may be required (24).

Therapies that address the dysbiosis associated with IBS may be added to this management plan (Figure 1). Dietary modifications and the use of probiotics may promote the growth of beneficial Lactobacilli and Bifidobacteria, alter intestinal gas production (CH_4_, H_2_) and modulate the immune response. Periodic short courses of the antibiotic rifaximin may also be effective in reducing the pro-inflammatory products of pathogenic bacteria and in re-establishing microbiota diversity, which may improve abdominal distension, pain and stool consistency (114–116).

Despite emerging evidence of the importance of dysbiosis in the pathophysiology of IBS, it should be noted that there is no consensus on how dysbiosis should be characterized. At present, eubiosis can only be defined retrospectively as changes to the microbiome that are presumed to underlie symptom improvement in individual patients rather than as a prospective goal of therapy. Preliminary attempts to develop a test for fecal dysbiosis have been unsuccessful (117), although fecal bacterial profiling may one day prove useful in predicting responders to treatments that target dysbiosis, such as a low FODMAP diet (118). Thus, modulation of the microbiome will require an individualized and empiric strategy of dietary modification, probiotics and/or use of nonsystemic antibiotics in an effort to modify the complex ecology of dysregulated microbiome–host interactions in IBS patients.
